# 2024: Year one—from inception to mass disruption of artificial intelligence in cardiology

**DOI:** 10.1093/ehjopen/oeae002

**Published:** 2024-01-17

**Authors:** Benjamin Marchandot, Antonin Trimaille, Olivier Morel

**Affiliations:** Division of Cardiovascular Medicine, Nouvel Hôpital Civil, Strasbourg University Hospital, 1 place de l’Hôpital, 67000 Strasbourg, France; UR 3074 Médecine Cardiovasculaire Translationnelle, Strasbourg University, Strasbourg, France; Division of Cardiovascular Medicine, Nouvel Hôpital Civil, Strasbourg University Hospital, 1 place de l’Hôpital, 67000 Strasbourg, France; UR 3074 Médecine Cardiovasculaire Translationnelle, Strasbourg University, Strasbourg, France; Division of Cardiovascular Medicine, Nouvel Hôpital Civil, Strasbourg University Hospital, 1 place de l’Hôpital, 67000 Strasbourg, France; UR 3074 Médecine Cardiovasculaire Translationnelle, Strasbourg University, Strasbourg, France; Hanoï Medical University, Vietnam

**Keywords:** Disruption, Artificial intelligence, Future, ChatGPT

Disruption refers to a significant and often abrupt change or disturbance that interrupts the normal course of events within a system, industry, or market. Disruption is intrinsically associated with innovation, and the introduction of new paradigms that first challenge and then replace existing practices. The timeline of disruption varies widely according to the concerned field (e.g. industry, technology, business etc.), and various external factors such as human acceptance. The general timeline for disruption is thought to be around 10 years (*Figure 1*).

**Figure 1:**
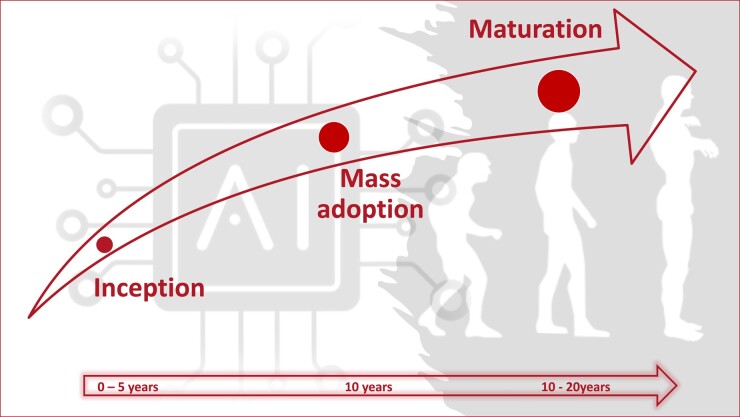
Graphical abstract illustrating the evolutionary concept of disruption over time.

The early stage or inception stage takes 0–5 years and refers to the development and introduction of a new technology, model, or idea. This stage encompasses the initial exploratory journeys, testing diverse applications, models, and strategic approaches. The discernible emergence of the newly paradigm first begins within a closed circle of initiates and then garners increasing attention in conjunction with novel entrants. Mass adoption typically occurs within 10 years. The disruptive technology has proven to be successful; the innovation has become widely accepted, and its impact is felt across the relevant field. Over time, the concept becomes fully integrated, establishes itself as the norm, and matures with the definition of standards, regulatory frameworks, and iterative improvements. The speed of disruption can be influenced by various factors, such as human acceptance, technological complexity, regulatory environment, and economic feasibility.

As it takes 10 years to go from medical school, through residency, and finally fellowship to become a cardiologist, new entrants in the field should be familiar with this timeline as it aligns with the timing of disruption. The year 2024 marks the first year after the ‘Artificial Intelligence (AI) Breakthrough’ in general cardiology. Our current dating system is known as the Anno Domini system, which is Latin for ‘In the Year of Our Lord.’ We refer to the year 2023 as the dawn of ChatGPT and all AI models in our lives and medical practice, reminiscent of the Muggle we were back then.

ChatGPT, a large language model, was made freely available by OpenAI in November 2022 and gained significant attention in 2023 (Year 0). It took only 5 days for ChatGPT to amass 1 million users, a feat achieved in comparison to 3.5 years for Netflix, 10 months for Facebook, 5 months for Spotify, and 2.5 months for Instagram. Using a PubMed search, there were only four references found for ‘ChatGPT’ in 2022, none before that, and ∼1766 by early December 2023. It compelled academic and publishing regulatory organizations to promptly establish new rules. Forrester Research forecasted that generative AI will constitute nearly 30% of the jobs lost to automation by 2030.^1^ Disruptive, isn’t it?

Good or bad, sooner or later, it is beyond the scope of this letter to evaluate the essence of the generative AI breakthrough in our general life. However, what can we forecast for cardiology? How will this disruptive model reshape general cardiology in the next 10 years? Muggle to generative AI yesterday; let us be a mage for a minute and forecast the cardiology weather in 10 years from now.

- Education and prevention are no longer an issue, as generative AI provides instant responses to patients according to latest recommendations.
- Digital stethoscopes with AI-supported analysis reduce the burden of cardiovascular disease detection across the globe.
- The burden of hypertension and associated complications has been drastically reduced with the widespread use of wrist-worn devices for blood pressure monitoring.
- Formal instruction in electrocardiogram (ECG) interpretation has almost been phased out in medical education, as fully automated analysis is now universally acknowledged for its unparalleled reliability, achieving a 100% accuracy rate.
- Echocardiograms are now exclusively performed by paramedics, as it only takes an instant to gather transthoracic views with fully guided acquisition through AI. Physicians only focus on the review of images in general cardiology.
- Smart sock tracking ankle oedema variation provides instant counselling to patients, indicating when to consult at heart failure (HF) ambulatory HF clinics.
- Advanced sensors designed for the detection of cardiac biomarkers are making notable strides, particularly within the population of HF patients, effectively easing the patient journey in ambulatory care.
- Smartwatch and ECG recording devices are widely adopted in the general population. The 2023-NOAH-AFNET 6—trial is no longer a matter of concern, as dedicated trials and cohorts have identified patients with very rare and short atrial arrhythmias who are at a high risk of stroke and benefit from anticoagulation.
- Smartwatch-derived ECG has been replaced by electronic skin patches that record acute myocardial infarction live, localizes you, and automatically sends you a first aid medical team as soon as possible. The nearest cath lab receives a notification, and the time from symptom onset to revascularization is no longer a matter of issue in the literature.
- Artificial intelligence and machine learning accelerate clinical trials by reducing the required number of participants, costs, and the time needed to process clinical trial data.

Disruption of AI is more or less pronounced according to specialties. The perception of replacement toward AI may preferentially affect radiologists nowadays^2^ or dermatologists, with visual inspection to identify suspicious pigmented lesions being supplanted by AI networks.^3^ The perception of being potentially replaced, rigorously challenged, and the need for more knowledge and a change in our attitude towards AI are becoming evident in cardiology. In light of the dynamic landscape of AI in cardiology, a nuanced perspective that considers both the potential benefits and challenges associated with its rapid development will be a crucial step in the coming months and years. Striking a balance between the apparent rapid advantages of innovation and staying mindful of the complexities, along with the necessity for rigorous scientific validation of their beneficial effects, will be imperative. While the accuracy, diagnostic performance, and accessibility of upcoming AI tools in cardiology are likely to surpass our human practice in a 10-year time frame, nothing will replace the importance of empathy, reassuring words, and a genuine smile for our patients. Even with AI on the horizon for general cardiology, the comfort of a steadying hand remains irreplaceable.

## Data Availability

No new data were generated or analysed in support of this research.
